# Comparison of Microstructure and Mechanical Properties of Scalmalloy^®^ Produced by Selective Laser Melting and Laser Metal Deposition

**DOI:** 10.3390/ma11010017

**Published:** 2017-12-23

**Authors:** Mustafa Awd, Jochen Tenkamp, Markus Hirtler, Shafaqat Siddique, Markus Bambach, Frank Walther

**Affiliations:** 1 Department of Materials Test Engineering (WPT), TU Dortmund University, 44227 Dortmund, Germany; jochen.tenkamp@tu-dortmund.de (J.T.); shafaqat.siddique@tu-dortmund.de (S.S.); frank.walther@tu-dortmund.de (F.W.); 2 Chair of Mechanical Design and Manufacturing (KuF), BTU Cottbus-Senftenberg, 03046 Cottbus, Germany; hirtler@b-tu.de (M.H.); bambach@b-tu.de (M.B.)

**Keywords:** Scalmalloy^®^, additive manufacturing, mechanical properties, damage mechanisms

## Abstract

The second-generation aluminum-magnesium-scandium (Al-Mg-Sc) alloy, which is often referred to as Scalmalloy^®^, has been developed as a high-strength aluminum alloy for selective laser melting (SLM). The high-cooling rates of melt pools during SLM establishes the thermodynamic conditions for a fine-grained crack-free aluminum structure saturated with fine precipitates of the ceramic phase Al_3_-Sc. The precipitation allows tensile and fatigue strength of Scalmalloy^®^ to exceed those of AlSi10Mg by ~70%. Knowledge about properties of other additive manufacturing processes with slower cooling rates is currently not available. In this study, two batches of Scalmalloy^®^ processed by SLM and laser metal deposition (LMD) are compared regarding microstructure-induced properties. Microstructural strengthening mechanisms behind enhanced strength and ductility are investigated by scanning electron microscopy (SEM). Fatigue damage mechanisms in low-cycle (LCF) to high-cycle fatigue (HCF) are a subject of study in a combined strategy of experimental and statistical modeling for calculation of Woehler curves in the respective regimes. Modeling efforts are supported by non-destructive defect characterization in an X-ray computed tomography (µ-CT) platform. The investigations show that Scalmalloy^®^ specimens produced by LMD are prone to extensive porosity, contrary to SLM specimens, which is translated to ~30% lower fatigue strength.

## 1. Introduction

Metal additive manufacturing processes opened new dimensions of technological freedom through an extensive capability of tailoring property and function of metallic structures. Selective laser melting (SLM) and electron beam melting (EBM) are the flagships of this new technology. The two processes that have similar layout involve micro welding of powder layers with a thickness of 30–50 µm and 100 µm for SLM and EBM, respectively [1]. The primary advantage of the latter two over the more traditional selective laser sintering (SLS) is the complete melting of powder particles in micro melt pools. The total melting results in a melt track with higher structural integrity [2]. To this end, SLM and EBM technologies can be applied to manufacture structures subjected to dynamic loadings. SLM and EBM have been used to titanium (Ti) alloys for the commercial grade 5 [3], and the biomedical variant of Eli Ti-6Al-4V [4], for foreseeable application in aerospace and medical implants industries. Titanium in its commercially pure status, manufactured by SLM, was proved imminently suitable for use in wear resistance applications. Further improvements were possible by the constitution of Nano-composite through SLM deposition with Ti-B particles. Consequently, hardness and elastic modulus were improved due to reinforcing particles [5]. Relative densities as high as 99.5% were achieved. The microstructure refinement by SLM was the imminent factor in comparison to laser engineering net shaping (LENS) concerning mechanical properties [6]. A study of Inconel 718 reported that microstructure is dependent on the cooling rate that is sensitive to scanning speed. The findings were supported by melting simulation using the finite volume method (FVM). Under variation of scanning speed, metallurgical porosity migrated to the surface of the component [7]. The degree of energy concentration in SLM qualified it for processing of aluminum (Al) alloys. The latter cannot be processed by EBM since preheating scans are already exceeding melting temperature of Al alloys (~667 °C). Additionally, electron beams cannot apply enough concentrated energy to break through the highly thermal-resistant ceramic phase of Al_2_-O_3_ that accumulates on Al surfaces exposed to the atmosphere, given the high-thermal-conductivity and beam reflectivity of Al surfaces [8]. Already available advanced Nd: YAG-laser-based SLM platforms can provide such a processing window, which was not possible for older CO_2_-laser-based SLM platforms [9].

Investigation of high-strength alloys with tailored properties through additive manufacturing has been an extensive topic of research. Production of Nano-composites based on 316L steel through SLM was studied to improve specific moduli, yield strength, hardness and wear resistance. Densification properties were reported to be dependent on TiB_2_ content [10], as well as scan speed and the net energy density. Grain size, orientation, and texture were possible to control through scanning parameters. Consequently, the compressive strength was improved due to grain refinement [11]. Tailoring of mechanical properties was possible by controlling heat dissipation directions using different scanning strategies and remelting. The proposed strategy allowed for the constitution of microstructure with controlled heterogeneity [12]. Scanning strategy also had a significant effect on the microstructure and precipitation behavior of 17-4PH stainless steel. Remelting enhanced relative density as well as part accuracy. Aging treatments afterward increased hardness up to 458 HV_0.30_ [13].

Although Al alloys are physically different to steel and Ti alloys regarding thermal conductivity, surface reflectivity, and melt viscosity, SLM-induced properties are similar. For eutectic AlSi12 and AlSi10Mg, high cooling rates resulted in the constitution of a fine 3D lamellar-dendritic network of a eutectic phase [14,15]. Morphology of the microstructure was equiaxed dendritic in the XY-plane and columnar dendritic in the perpendicular building plane. Thus, mechanical property anisotropy is induced. A byproduct was supersaturation of Si particles in the Al matrix [16], as well as a hardening phase of Mg_2_-Si in AlSi10Mg. This resulted in an increased tensile strength by almost ~300%, concerning sand-cast or die-cast Al alloys of the same composition. At melt pool boundaries, laser tracks overlap. Hence, a coarser spheroidized microstructure is constituted [17]. During damage and failure analysis, under quasi-static loading, dendritic arms were identified as load carrying elements of the Al-Si eutectic structure. Failure is preceded by breakage of the dendritic arm at the weakest link, followed by delamination of the Al matrix, and ultimately crack initiation, propagation, and fracture [18]. A side-effect of high-cooling rates is an accumulation of remnant porosity. Concerning quasi-static loading, relative density in the range of ~99% or higher did not reduce tensile strength. The effect of remnant porosity in this mode of loading was limited to a decrease in fracture strain [19]. Full plastification of the gage length was defined by formation of early cracks at critical pores. Thus, coalescence of multiple cracks resulted in a rapid fracture and lower fracture strains concerning conventional alloys of the same composition. The interaction between scanning direction and flow of inert gas had an effect on AlSi10Mg component quality in terms of tensile strength. Scanning in the direction of gas flow limited the balling and splashing effects resulting from melt pool instability. Hence, finer structures were possible to obtain, which had a positive effect on mechanical properties [20]. 

For Al alloys, surface conditions from the as-built state were more influential in HCF behavior. Stress-relieved AlSi10Mg showed enhanced fatigue strength due to the increased ductility of the modified microstructure [19]. Crack initiations from surface and subsurface defects were reported to induce significant scatter for AlSi10Mg and AlSi12. In both alloys, platform heating (PH) resulted in enhanced energy density, lower remnant porosity, and reduced fatigue scatter [21]. Hot isostatic pressing (HIP) of AlSi12 leads to a decrease of both quasi-static and fatigue strength because of alteration of the morphology of the eutectic structure as Si particles agglomerate beyond cohesiveness. The reported strength was still higher than the conventionally manufactured cast-alloy with reduced fatigue scatter [21]. In HCF and VHCF, specimens with PH experienced fatigue behavior dominated by resistance to crack initiation rather than propagation. Thus, failure was dominantly initiated from the surface in contrast to specimens without PH [22]. However, side-effects of porosity were reported in the role of a crack-blunting mechanism by locally reduced stress intensity factor. The effect was proved by a crack propagation–based fatigue life calculation. It also highlighted that plastic zones on the crack flanks can be employed to a crack-dampening effect [23]. Hybrid structures of SLM (AlSi12) and wrought alloys (EN AW-7020) for fatigue loading applications were reported feasible with fatigue strength of ~80 MPa at 10^9^ cycles [24]. A study of the interaction between the mesostructure and microstructure of AlSi12 suggested that the first damps the rate of crack propagation while the latter accounts for strength, hence, crack initiation resistance. A side-effect is a significant anisotropy in the as-built status [25]. Comparison between cast AlSi10Mg from the view of fatigue properties, in the casting status or from SLM, suggested common effects of oxides that precipitate from metal vapor. The oxide residues have an effect on damage initiation mechanisms concerning microscale deformations [26].

Wrought alloys belonging to the high-strength category of age-hardening alloying systems such as Al-Zn and Al-Cu are of great interest to the industry. Unfortunately, the latter ones proved unsuccessful to process using SLM because of development of hot cracks [27]. Hot tearing left the as-built structure at less than ~30% of the strength of conventional alloys of the same designation [28]. However, composition changes by addition of Si [29], Zn [30], and Zr [31], were favorable in the elimination of hot cracks. Thus, hardness and tensile properties were improved compared to wrought alloys of the 7xxx series. Hardness Vickers was reported to be 219 ± 4 HV_0.05_ [30]. Tensile strength increased to ~417 MPa by adding Zr [31]. In a similar context, APWorks of Airbus group adopted a fundamentally different approach to SLM of high-strength Al alloys. The Al-Mg-Sc alloy has been in development for two decades. Earliest reports of tensile strength were more than 420 MPa [32,33]. Also, the alloy was well processable in casting, forming, and welding [34]. A study on the effect of adding Sc to hypoeutectic and hypereutectic Al alloys found that a refinement effect is induced on the latter rather than the first. With the cooling of the ingots, Sc is supersaturated in the solid solution. Thus, strength is significantly increased with age treatments, as micro segregation develops [35]. Such findings materialized in the work of APWorks as second-generation Al-Mg-Sc alloy was developed as Scalmalloy^®^ for SLM processing. Early studies report successful processing of Scalmalloy^®^ using SLM. Relative density accomplished was well more than 99% at higher energy densities typical of other Al or Ni-based alloys [36]. The principle strengthening mechanism observed in microscopy was supersaturation of Sc particles as well as precipitation of Al_3_-Sc phase which pins grain boundary and hinders dislocation gliding, giving rise to superplastic material flow [37]. Concerning Scalmalloy^®^, the reported data of mechanical properties of the new alloy are less, with no fatigue data in the noticeable literature. In this study, Scalmalloy^®^ was successfully processed using SLM and laser metal deposition (LMD). LMD can be considered an alternative to SLM due to larger deposition rates which is relevant to large parts. Al 7050 has been recently processed using LMD with more than 99% relative density [38]. Processing AlSi12 using LMD resulted in ~100% dense parts with a tensile strength of 225 MPa and 9 × 10^−2^ fracture strain [39]. 

This paper aims at the investigation of mechanical strength of Scalmalloy^®^ in quasi-static and fatigue modes of loading such that a subsequent qualification to aerospace industry is possible. Damage mechanisms investigated are highly relevant for further potential enhancement through aging treatments. In parallel, a fatigue strength characterization scheme is presented based on finite-element (FE) analysis and Monte-Carlo simulation. Currently-existing models do not consider variation in fatigue strength, which is a typical characteristic of additively manufactured structures [23]. For highlighting the significance of the newly developed alloy, AlSi10Mg, the closest to Scalmalloy^®^ in composition, is subjected to similar conditions of loading and damage.

## 2. Materials and Methods

### 2.1. Experimental Setup

The materials investigated in this study are two alloys in four batches according to Table 1. Batches A and B were manufactured using the SLM process at Concept Laser with optimum scanning parameters in two different orientations. Specimens of batch A were built parallel to the building platform, while batch B was built perpendicular to the platform. 

Scalmalloy^®^ of APWorks was manufactured in two batches C and D. The chemical composition of Scalmalloy^®^ can be found in Table 2 as given by APWorks and Citim. Batch C was manufactured using LMD from a powder with approximate particle size of ~15–115 µm with an average size of 92.5 µm, parallel to the building platform. A TruCell 7040 system (Trumpf, Germany) with a laser power of 4 kW was used under Argon atmosphere. The feed rate and powder flow rate were 0.75 m/min and 2.7 g/min respectively. Batch D was manufactured using an SLM 280 machine of SLM solutions perpendicular to the building platform. The maximum particle size of the powder was 45 µm. The SLM process was conducted with a laser power of 500–700 W at a speed of 1200–1700 mm/s. The laser beam diameter was 60 µm.

#### 2.1.1. Structure and Morphology Analysis

**Figure 1:**
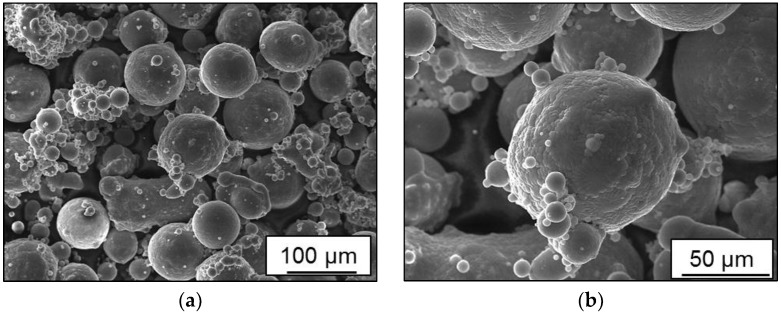
Powder morphology of Scalmalloy^®^: (**a**) 200X; (**b**) 500X.

The current study aims to establish structure-property relationships for AlSi10Mg and Scalmalloy^®^ for SLM and LMD processes. Hence, structure and morphology of available Scalmalloy^®^ powder, used in batch C, was investigated in SEM system of TESCAN. In Figure 1a, an overview of the non-fresh/used powder morphology is given. A wide distribution of particle size, as well as morphology, can be observed. Powder particles are experiencing significant irregularities due to formation of satellite particles in the reused powder. A closer view of satellite particles can be found in Figure 1b. Such morphological characteristics negatively influence powder flowability and melt pool viscosity in case of using recycled powder. Consequently, keyhole defects and metallurgical porosity are expected, depending on the given energy density per unit volume of the melt pool. Defects induced by additive manufacturing processes have a detrimental effect on the fatigue strength. In comparison to work done in [20], the influence of gas flow versus scanning direction was not considered for the study. The whole process setup differences between SLM and LMD were compared concerning fatigue properties. Since deposition of single-pure alloy per component is considered, densification problems due to mixing heterogeneities [10,11,12] were not experienced in this work.

**Figure 2:**
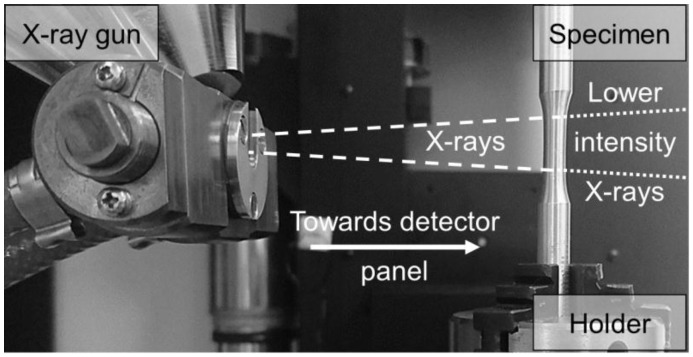
The experimental setup of X-ray microcomputed tomography (µ-CT) for scanning fatigue specimens.

The defect status can be used to predict fatigue lifetime when characterized by a non-destructive testing method such as µ-CT. For property-defect correlation and fatigue lifetime prediction, selected fatigue specimens were scanned using X TH 160 system of Nikon Metrology. The experimental setup is illustrated in Figure 2. An X-ray gun of 160 kV generates a cone-beam of X-rays that are projected at the gage length of a cylindrical fatigue specimen mounted on a concentric holder. The contrast of grey values passing through the specimen is collected at the detector. The specimen was rotated 360° with a step of 0.22° such that 1583 projections were generated. The superposition of the projections is used to reconstruct a 3D volume for defect analysis.

#### 2.1.2. Mechanical Properties

Specimens of batches A-D were subjected to a tensile test on an Instron 3369 machine with a load cell of 50 kN using an extensometer with a gage length of 10 mm. Two types of stress-controlled fatigue tests were applied to all batches. The first is the load increase (“cyclic tensile”) test, where the specimen is subjected to a stress amplitude that is increased as a function of time with a constant ramp. Hence, the cyclic response of a material can be monitored against a wide range of stresses, and a cyclic-tensile fracture-stress can be evaluated. Following the reaction of the material against the stress ramp can be used to detect critical stress amplitudes where new mechanisms of fatigue damage get activated concerning fatigue regime from HCF to LCF. The second type is a constant-stress amplitude fatigue test at selected levels based on the damage response in the load increase test. The procedure combining both forms is used as a high-throughput procedure for the characterization of fatigue strength and screening of disqualified materials.

**Figure 3:**
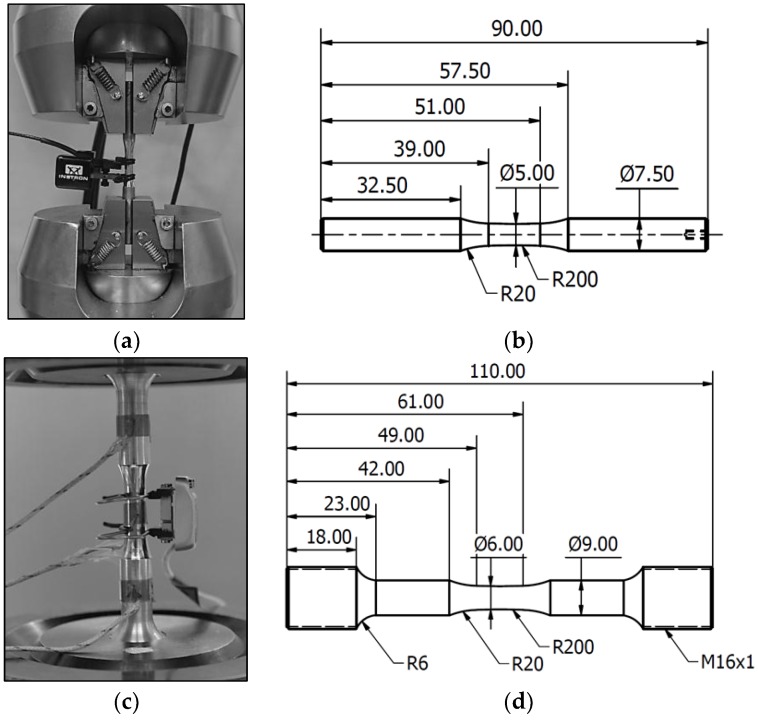
Experimental setup for mechanical testing: (**a**) Servohydraulic testing system; (**b**) Specimen geometry for batches A–D; (**c**) Resonance testing system; (**d**) Specimen geometry for batch D.

Batches A–D were subjected to the load increase test with the setup shown in Figure 3a and a specimen geometry of Figure 3b using a servohydraulic Instron 8872 system with a 10 kN load cell.

**Figure 4:**
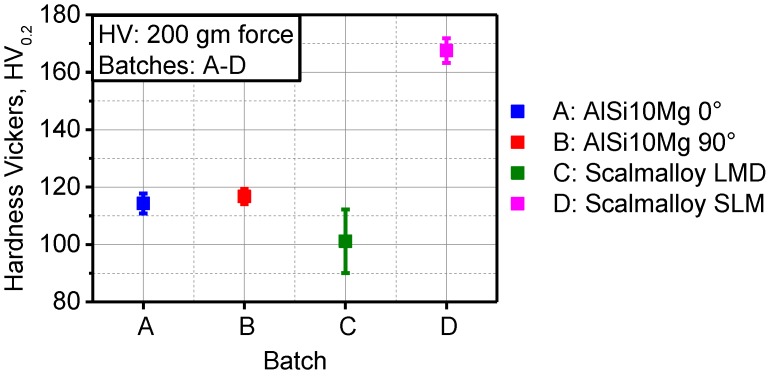
Microhardness measurements according to Vickers with 200 g-force of batches A–D.

The starting stress amplitude was set to 30 MPa, and the loading ramp was 10 MPa/10^4^ cycles, at a frequency of 20 Hz. During the constant amplitude testing phase, batches A–D were tested at 140 and 120 MPa using the same testing system, frequency, and specimen geometry. In batch D, additional constant amplitude testing was carried out at a Rumul Testronic machine with the frequency of 68 Hz at stress levels of 140, 120, 100, and 80 MPa. The setup is shown in Figure 3c and specimen geometry in Figure 3d. Using a microhardness measurement system Shimadzu HMV-G21, indentations with 200 g-force load were applied. The mean and standard deviation of five measurements per batch are reported in Figure 4, where batch D experienced the highest hardness in comparison to batch C with the lowest hardness. Batches A and B were comparable. Hardness measurements are to be correlated with tensile strength and densification behavior during alloy deposition. Such significant difference between batches C and D in the measured hardness can stem from two sources. The first is that cooling rate in LMD (batch C) is much lower than cooling rate in SLM. Lower cooling rate induces coarser microstructure and consequently lower microhardness. The second reason is the extensive multiscale porosity which remained in batch C due to LMD process. Remnant porosity induced compliance of the microstructure, hence microhardness is reduced.

### 2.2. Fatigue Lifetime Calculation

#### 2.2.1. Finite-Element Analysis

**Figure 5:**
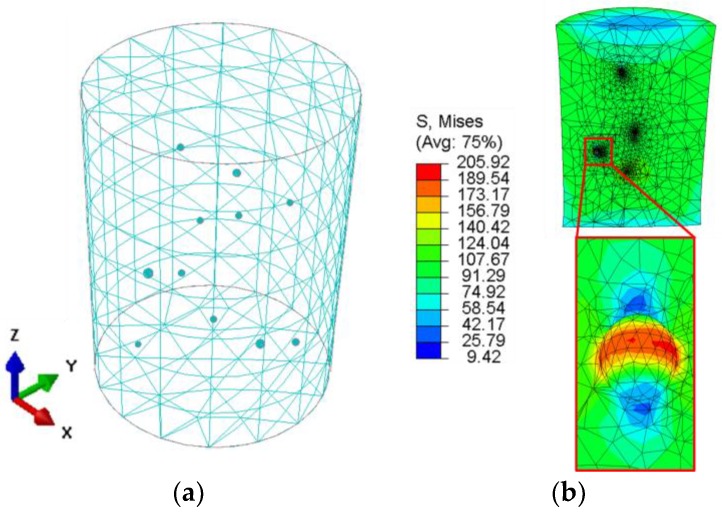
Finite-element analysis of batch C (Scalmalloy^®^ LMD) under 100 MPa of stress amplitude: (**a**) Model; (**b**) Z-plane cross-sectional peak-stress distribution, around pores under uniaxial cyclic loading.

Finite-element modeling is used in this section to calculate stress distribution of stable cyclic response within a specimen subjected to constant amplitude loading. Critical pores from the CT scan are selected based on the pore size as well as the principle of statistical significance. The material law was derived based on the reaction of material during the load increase (“cyclic tensile”) test by fitting experimental data. A combined isotropic and kinematic hardening material law was employed. The solution employs an indirect conversion of Newton-Raphson scheme in Abaqus 6.12 (Simulia, RI, USA) simulation software [40]. The applied cyclic frequency constitutes a Fourier series representation of displacement at a specific iteration.

(1) $$\bar{u}\left(t\right)=u_{o}+\overset{n}{\underset{k=1}{\sum }}\left[u_{k}^{s}\;\sin\left(k\omega t\right)+u_{k}^{c}\;\cos\left(k\omega t\right)\right]$$

where n is the number of Fourier terms, ω = 2π/T the angular frequency, and u_o_, u_k_^s^, u_k_^c^ are displacement coefficients. Fourier series representation is a more efficient representation of the variable of the boundary value problem since a fewer number of terms can be used without losing accuracy of the solution. Comparison of the obtained solution follows iteratively until the residual diminishes.

(2) $$\bar{R}\left(t\right)=R_{o}+\overset{n}{\underset{k=1}{\sum }}\left[R_{k}^{s}\;\sin\left(k\omega t\right)+R_{k}^{c}\;\cos\left(k\omega t\right)\right]$$

where R_o_, R_k_^s^, R_k_^c^ are residuals corresponding to displacement coefficients u_o_, u_k_^s^, u_k_^c^, respectively. Stress distribution using this FE analysis is exported for post-processing and lifetime calculation. The post-processing which was done in a self-developed tool was carried out to calculate Dang-Van threshold stresses as it will be shown in the next lines. The FE model for batch C is shown in Figure 5a, was generated from the µ-CT results, and the cross-sectional stress distribution is shown in Figure 5b.

#### 2.2.2. Statistical Modeling

**Figure 6:**
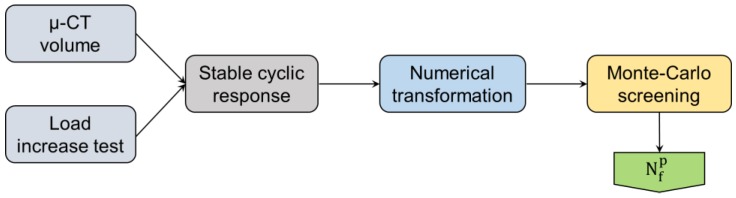
The graphical algorithm of the fatigue lifetime calculation model.

Statistical modeling aims to account for fatigue scatter caused by the uncertain generation of defects. Unexpected generation of defects leads to uncertainty about resulting properties; thus process setup is not able to reproduce the properties. The first phase of Monte-Carlo simulation is to screen results from the FE analysis. Screening is applied to this study by application of a multiaxial fatigue failure criterion of Dang-Van.

(3) $$\sigma_{\mathrm{DV}}=\tau_{\max}+{\varphi \;P}_{\max}$$

where $\sigma_{\mathrm{DV}}$ is the Dang-Van amplitude, $\tau_{\max}$ is the maximum shear stress, φ is the sensitivity factor, and $P_{\max}$ is the maximum hydrostatic pressure. Finite elements developing a stress less than the Dang-Van amplitude are excluded from the analysis. They are not expected to be failure initiation sites. Thus, the probability of failure at such elements vanishes. Elements which surpass Dang-Van filtering are used as a discrete probability density function. Using the maximum-likelihood method, a Weibull-based fatigue strength distribution is established

(4) $$f\left(x;\;\alpha ,\;\beta \right)=\;\{\begin{array}{ccc} \alpha \;\beta \;x^{\beta -1}{\;e}^{-{\alpha x}^{\beta }} & , & \;x>0\;\\ 0 & , & \;\mathrm{elsewhere} \end{array}$$

where x is a random variable, α is Weibull scale parameter, and  β is Weibull shape parameter. From this density function, random draws for Monte-Carlo simulation are generated following a numerical transformation of stress distribution

(5) $$x_{i}^{\prime }=b+\;\frac{x_{i}-x_{\min}}{x_{\max}-x_{\min}}\;\left(a-b\right),$$

where $x_{i}^{\prime }$ is a transformed random variable, a and b are the limits of transformation interval. Iterations continue until the lower bound fatigue strength is detected with maximized probability. From this point, all calculated fatigue lifetime values according to probabilistic Basquin equation

(6) $$N_{f}^{p}=\;{\left(\frac{b_{n\;}\sigma_{a}}{P_{r}\;K}\right)}^{1/b_{m}},$$

are considered valid where $N_{f}^{p}$ is a probabilistic fatigue life, $b_{n}$ is Monte-Carlo quadrature, $P_{r}$ is the probability correction factor, K is the fatigue strength coefficient, and is the fatigue strength exponent. The analysis continues until the probability of the calculated lifetime converges to zero. Thus, upper bound fatigue lifetime is reached. Further details on the simulation procedure can be found in [23,41], where the derivation of equations and assumptions were in the scope of the cited papers. An overview of the flow of information during this algorithm can be seen in Figure 6. First, the µ-CT volume is represented as an FE model where material law from the load increase test is applied to obtain a stable cyclic response. Numerical transformation based on the maximum-likelihood method follows. From it, random draws are generated for Monte-Carlo simulation.

## 3. Results and Discussion

### 3.1. Defects and Microstructure

**Figure 7:**
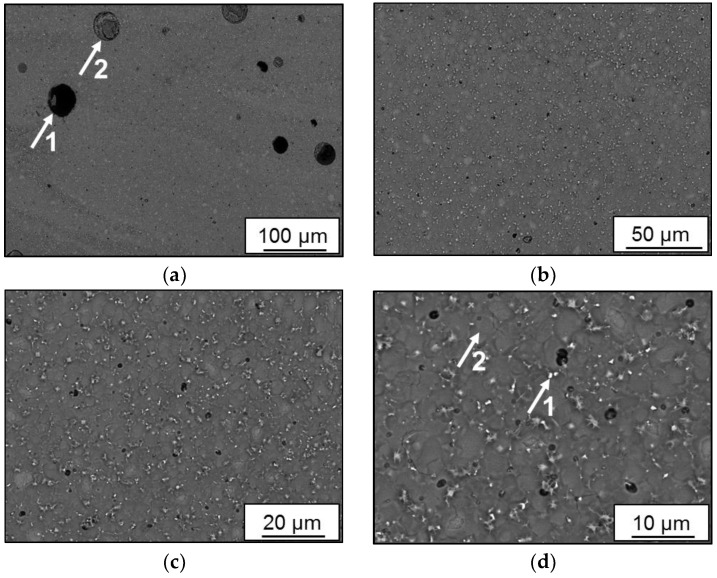
Scanning electron microscopy (SEM) micrographs in melt tracks of batch C (Scalmalloy^®^ LMD): (**a**) 200X; (**b**) 500X; (**c**) 1000X; (**d**) 2000X.

An overview of the microstructure in the backscattered electron (BSE) mode of batch C can be seen in Figure 7a. Specimens were etched using a solution of NaOH with a concentration of 4%. Two types of pore morphologies can be observed. Pore 1 with the smooth inner surface is a metallurgical pore that is generated by an excessive energy density and balling phenomena. Pore 2 is a pore that is created due to inhomogeneity of the melt pool as an unmelted particle is captivated inside. Figure 7a indicates the formation of excessive porosity on a low scale of the microstructure. Pore morphology will be confirmed by a discussion of µ-CT results. 

Figure 7b shows an overview of microstructure inside the melt track where homogeneity of the microstructure can be observed. Finely grained equiaxed cells are constituted in Figure 7b, while precipitation on the grain boundary is apparent in Figure 7c. Precipitation of particles of the hard Al_3_-Sc phase is the primary strengthening mechanism in this designed alloy. They pin grain boundaries and accommodate plastic deformation when material flows under loading. Hardening phase particles are indicated in Figure 7d at arrow 1. An equiaxed grain is shown by arrow 2, apparently clear of Al_3_-Sc precipitation.

**Figure 8:**
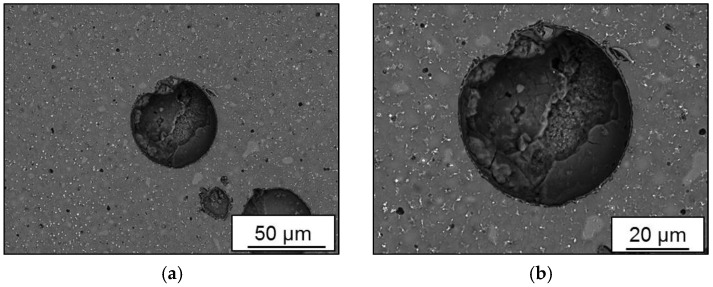
Pore morphology in batch C (Scalmalloy^®^ LMD): (**a**) 500X; (**b**) 1000X.

A closer view of pore morphology with surrounding microstructure is given in Figure 8 for batch C. The overview in Figure 8a indicates coarser grains as compared in the direction away from the pore. Coarsening of grains can be attributed to the concentration of heat and extended cooling cycle which is the main reason behind the generation of this metallurgical pore. In Figure 8b, a variation of brightness of individual grains, distributed coaxially concerning the pore, indicates different composition and a higher percentage of light elements, such as Si, in these grains. The bright phase of Al_3_-Sc is accumulated at the grain boundaries.

**Figure 9:**
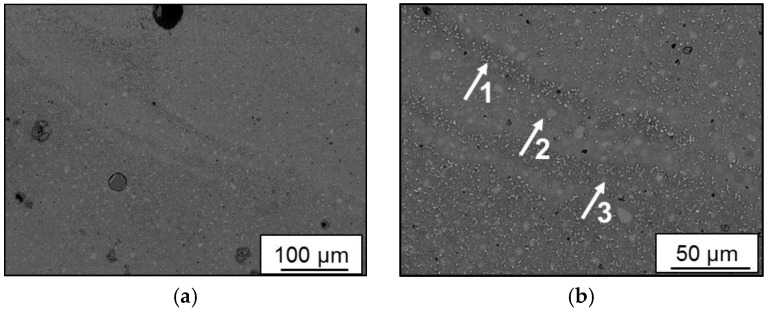
Melt pool boundaries in batch C (Scalmalloy^®^ LMD): (**a**) 200X; (**b**) 500X.

Melt pool boundaries of batch C are indicated in Figure 9a as an overview where several grain morphologies can be observed indicating fields of different cooling rates. Formation of porosity alongside the pool boundaries can also be seen. In Figure 9b, grades of boundary morphology are noted in the direction away from pool center as indicated by arrows 1, 2, and 3. Alternating cycles of heating and cooling, as layers are deposited, have influenced grain morphology as fine grain/high precipitation at arrow 1 is formed. At arrow 2, there is coarse grain/low precipitation, saturated with lighter brighter elements, since higher cooling rates induced a super cooling effect at this boundary interface. At arrow 3, a similar morphology to arrow 1 is observed since lower cooling rate allowed agglomeration of precipitation particles, unlikely to be cohesive with the Al matrix. Preprocess versus post-process segregation was not observed in this study.

**Figure 10:**
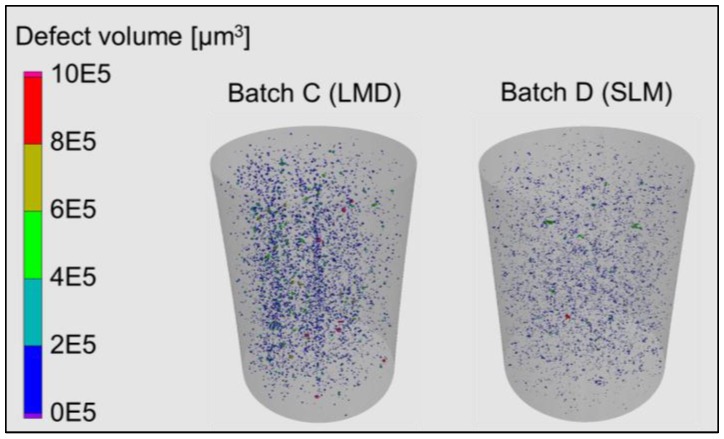
Defect distribution from µ-CT to batches: C (Scalmalloy^®^ LMD) and D (Scalmalloy^®^ SLM).

An overview of defect status of a selected fatigue specimen per batches C and D is given in Figure 10. Using the µ-CT setup of Figure 2, the reconstructed volumes were evaluated in VGStudio Max 2.2 (Heidelberg, Germany) based on a threshold probability of 1.0 and minimum defect size of 1 voxel. As can be seen in Figure 10, batch C developed excessive metallurgical porosity indicating excessive energy density application. In contrast to batch C, batch D developed keyhole pores indicating less energy density than the optimum was applied. Consequently, the relative densities for batches C and D were 99.65% and 99.96%, respectively. For batches A and B, the relative densities were 99.79% and 99.75%, respectively. It can also be observed that defect volumes in batch C are higher leading to less structural integrity, early crack initiation, and premature fracture. By taking such considerations into account, attempts to enhance densification through remelting, in the LMD process, will be considered in the outlook, as it was shown in the introduction [13].

### 3.2. Fatigue Strength

**Figure 11:**
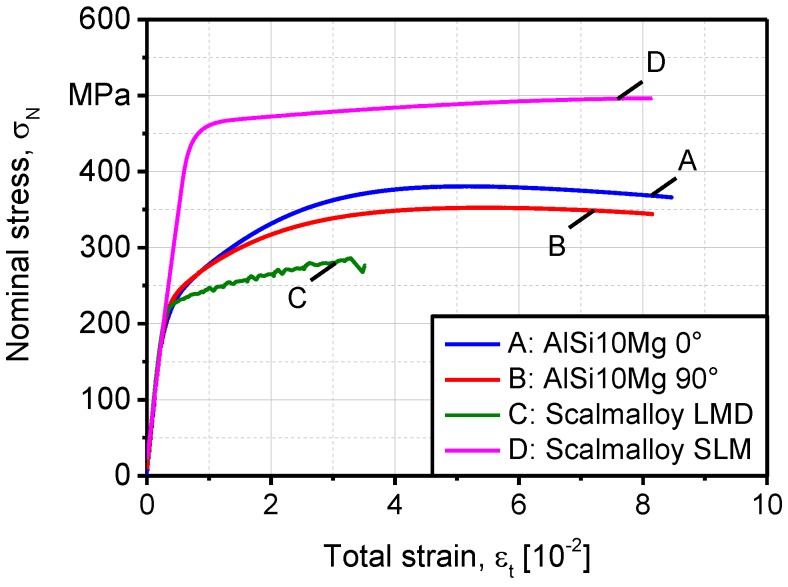
Quasi-static mechanical properties of batches A–D.

Characterization of fatigue strength of Scalmalloy^®^ is the primary aim of this study, even though several structural and property characteristics of the material of interest are investigated. The reason behind is that all types of material behavior are correlated to each other concerning fatigue regime under consideration. It is well-perceived that materials with better tensile strength properties are stronger in LCF regime. On the contrary, this is not applicable for HCF and very-high cycle fatigue (VHCF) regimes. In these regimes, the load increase test is regarded as more relevant. In Figure 11, quasi-static tensile properties of batches A–D are presented. The strength of batches A and B were comparable, although it was clear that specimens built at 0° enjoy higher tensile strength due to the application of less SLM layers during deposition, leading to higher structural integrity. 

Interlayer defects are potential failure initiation spots, especially when several cavities coalesce under loading. The difference in tensile strength, between batches C and D, is more than ~1.5 times in favor of batch D. The main reason for this is the less structural integrity of batch C due to excessive porosity. The latter induced a strain aging effect and disturbed the plastic flow causing premature failure. Less strength is also due to incoherent precipitation of secondary phases. In batch D, the SLM process, with its higher cooling rates, imposes a super finely grained structure (~0.4 µm dendrite thickness) with constitutional undercooling of mush constituents. The result is an Al matrix, supersaturated with extremely fine Al_3_-Sc and Si particles, that lead to superior plastic flow properties. Given these results, an interesting comparison is developing between Scalmalloy^®^ and Al_85_Nd_8_Ni_5_Co_2_ alloy, since the latter developed a compressive strength of ~1.00 GPa [42].

**Figure 12:**
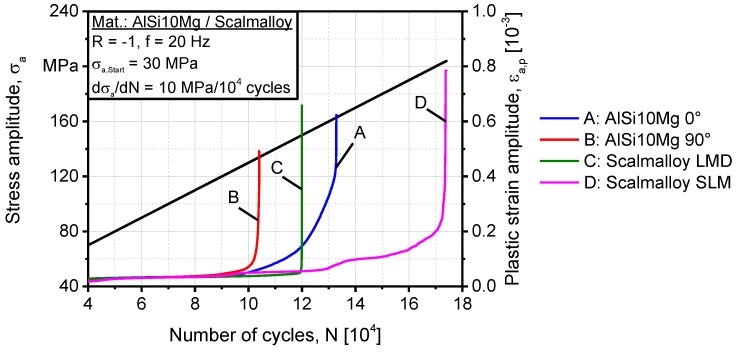
Plastic strain response of batches A–D in a load increase test that is used as input to the finite-element (FE) simulation.

The plastic strain response of batches A-D is shown in Figure 12 as a function of the number of cycles and the stress amplitude. For AlSi10Mg of batches A and B, batch A was also stronger as it has been the case in tensile testing. Both batches experienced damage initiation response at ~120 MPa of stress amplitude. Nevertheless, batch A was more tolerant to plastic damage accumulation as the stress amplitude was increased and the evolution of damage was a gradual change of slope of the plastic strain amplitude until a vertical asymptote was reached, eventually fracture. In case of batch B, the damage was abrupt resulting in a fracture stress of ~134 MPa in comparison to ~163 MPa for batch A. Although batch C has higher tensile strength than batch B, it experienced a fracture stress of ~150 MPa in the load increase test without the evolution of plastic damage. The damage evolution was probably suppressed by the existence of excessive porosity that entangles plastic deformation. The improved strength of batch C compared to batch B in the load increase test is due to fewer deposition layers as batch C is built at 0° orientation. In batch D, plastic damage evolution was more progressive, with several damage mechanisms activated in response to the increasing stress amplitude. The first initiation of damage was detected at ~120 MPa, where the damage accumulation exceeded the extent of single grains. Beyond this load level, accumulation of plastic damage progressed on the scale of several grains which experience sliding of boundaries until a stress level of ~160 MPa is reached. This helps understand the effect of Al_3_-Sc phase from the view of microstructure-property relation. Beyond this level, the combined effects of grain plastic-damage, multi-crack initiation, as well as porosity, accelerated and intensified damage leading to the final evolution and fracture at a stress amplitude of ~203 MPa. Significant differences between quasi-static and cyclic properties of batches B and C can be attributed to microstructure mechanics rather than mesoscale damage accumulation. Under cyclic loading, kinematic hardening attributes seemed to be more active in increasing crack initiation energy, thus prolonging life. A micromechanics study should follow to compare cyclic hardening behavior of a Si-saturated matrix in comparison to a Sc-saturated matrix.

**Figure 13:**
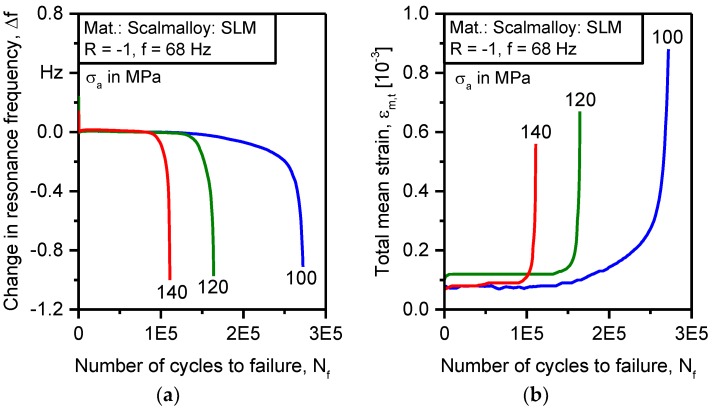
Specimen response of resonance testing for batch D (Scalmalloy^®^ SLM): (**a**) Change in resonance frequency; (**b**) Total mean strain.

Evolution of plastic damage in batch D was complex and included several stages, as well as load-dependent activated mechanisms. Constant amplitude testing on the Rumul Testronic machine was employed to separate damage mechanisms at selected stress levels. The principle of the system relies on the no-phase-shift resonance of two masses between which the specimen is clamped. The in-phase setup gives a possibility to correlate the change in resonance frequency to strain damage accumulation. The damage parameter is the total mean strain which represents the shift of the hysteresis centroid as plastic damage accumulates. The indirect relation between the resonance frequency and total mean strain is established by monitoring the dynamic stiffness of the specimen. Figure 13a shows a change in resonance frequency as a function of the number of cycles to failure for batch D. During loading at 140 and 120 MPa, the specimens’ reactions show no evolution of change in resonance frequency as well as the total mean strain of Figure 13b. At 100 MPa test, the development of frequency change and the total mean strain was significant. Since the change in resonance frequency is related to the dynamic stiffness of the specimen, it can be deduced that damage below 100 MPa is confined to single grains at the early life of the specimen until short cracks are initiated, and damage progresses beyond separate grains. At this point, the evolution of damage starts. 

**Figure 14:**
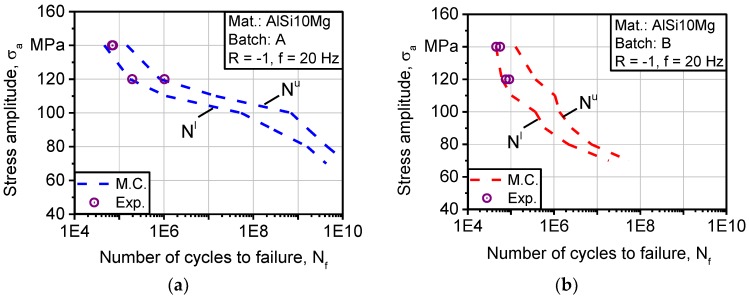
Comparison between calculated and experimental fatigue strength of batches: (**a**) A (AlSi10Mg 0°); (**b**) B (AlSi10Mg 90°).

Figure 14a depicts a comparison between experimental and calculated fatigue lifetime of batch A. The obtained experimental results lie within the expected boundaries of fatigue life at 140 and 120 MPa. At 140 MPa, two experiments coincided with the lower bound of fatigue life. At 120 MPa, one experimental point lies at the lower bound while the other lies at the upper bound. In batch B, in Figure 14b, experimental results are also coinciding with calculated fatigue life, where all lie at the lower bound for 140 and 120 MPa. By comparing modeling data, it can be deduced that fatigue strength of batch A is higher than that of batch B. The improvement in strength of batch A is more pronounced as the number of cycles increases toward HCF and VHCF, concerning lower stress amplitudes.

**Figure 15:**
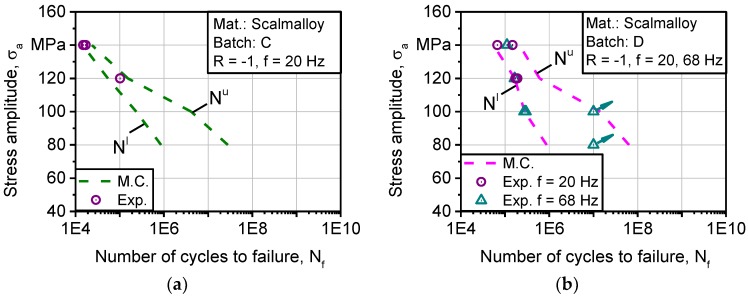
Comparison between calculated and experimental fatigue strength of batches: (**a**) C (Scalmalloy^®^ LMD); (**b**) D (Scalmalloy^®^ SLM).

Figure 15a depicts a comparison between experimental and calculated fatigue lifetime for batch C. The obtained results lie within the predicted range for stresses of 140 and 120 MPa. At 140 MPa, both experimental points lie at the lower bound. At 120 MPa, only one specimen was tested which coincided with the upper bound. As the number of cycles increases concerning applied stress amplitude, modeling data concludes that scatter of fatigue strength increases significantly due to the sensitivity of fatigue strength to defects in HCF regime. The scatter indicates that no repeatability of properties is possible with the process setup of batch C. In Figure 15b, validation tests were carried out at 140, 120, 100, and 80 MPa, with a limiting number of cycles of 10^7^. All experimental points coincide with the calculated boundaries except at the two run outs of 100 and 80 MPa of stress amplitude. It is highly probable that they would have exceeded upper bound if the test had continued. Nevertheless, the fatigue strength of batch D was higher especially in LCF to HCF. With high scatter in HCF towards VHCF, process repeatability is not high. The number of fatigue specimens used for establishing S-N curves corresponds to the experimental points plotted in Figure 14 and Figure 15.

## 4. Conclusions

In Scalmalloy^®^, the metallurgical features that are responsible for the improved strength were investigated. The supersaturation of light elements like Si in the Al matrix and precipitation of hard phases like Al_3_-Sc on the grain boundaries are the principle strengthening mechanisms. This qualified selective laser melted Scalmalloy^®^, built at 90°, to reach a tensile strength of 490 MPa in comparison to 380 MPa for AlSi10Mg, built at 0°. Scalmalloy^®^ produced by laser metal deposition has lower tensile strength, due to the coarser grains, resulting from the lower cooling rates of this process, in comparison to selective laser melting. Additionally, laser metal deposition of Scalmalloy^®^ induced extensive metallurgical porosity that activated a fracture mechanism based on the coalescence of defects rather than plastic damage. These structural features also resulted in a reduced hardness in comparison to Scalmalloy^®^ produced by SLM and AlSi10Mg. The proposed mechanism behind accelerated failure in batch B (90°), in comparison to A (0°), is that in this fatigue loading condition more interlayer defects are subjected to a perpendicular load.

Under cyclic loading, the superposition of several plastic damage mechanisms was detected using the load increase test. The separation of mechanisms was possible by monitoring damage parameters at several load levels of constant amplitude loading. It was revealed that in low-cycle fatigue, the cyclic damage is dominated by extensive plastic damage beyond grain boundaries. In high-cycle fatigue, the initial damage is confined to within grain boundaries until cracks are initiated beyond the boundaries of the grains. The coalescence of the short cracks induces extensive fields of plastic damage that involve several grains. Thus, specimen stiffness is reduced significantly.

The results of fatigue life predicted by Monte-Carlo simulation was found to be consistent with the experimentally determined fatigue life. Comparison of modeling results concluded that fatigue strength of selective laser melted Scalmalloy^®^ was superior to laser metal deposition of the same alloy. The lower and upper bound of Woehler curves indicated high scatter towards high-cycle fatigue. From a structural reliability point of view, the process repeatability is not precise. Further investigations will focus on enhancing process repeatability as well as microstructure and property control to resist damage mechanisms in high-cycle and very high–cycle fatigue.

## Figures and Tables

**Table 1:** Study design for comparison of mechanical properties of AlSi10Mg and Scalmalloy^®^.

Batch	A	B	C	D
Alloy	AlSi10Mg	AlSi10Mg	Scalmalloy^®^	Scalmalloy^®^
Process	SLM	SLM	LMD	SLM
Build orientation	0°	90°	90°	90°

**Table 2:** The chemical composition of Scalmalloy^®^.

Element		Mg	Sc	Zr	Mn	Si	Fe	Zn	Cu	Ti	O	V
wt %	(min)	4.00	0.60	0.20	0.30	0.00	0.00	0.00	0.00	0.00	0.00	0.00
	(max)	4.90	0.80	0.50	0.80	0.40	0.40	0.25	0.10	0.15	0.05	0.05

## References

[1] Gong H., Rafi K., Gu H., Janaki Ram G.D., Starr T., Stucker B. (2015). Influence of defects on mechanical properties of Ti-6Al-4V components produced by selective laser melting and electron beam melting. Mater. Des..

[2] Song B., Dong S., Zhang B., Liao H., Coddet C. (2012). Effects of processing parameters on microstructure and mechanical property of selective laser melted Ti-6Al-4V. Mater. Des..

[3] Qiu C., Adkins N.J.E., Attallah M.M. (2013). Microstructure and tensile properties of selectively laser-melted and of HIPed laser-melted Ti-6Al-4V. Mater. Sci. Eng. A.

[4] Amin Y.S., Ahmadi S.M., Wauthle R., Pouran B., Schrooten J., Weinans H., Zadpoor A.A. (2015). Relationship between unit cell type and porosity and the fatigue behavior of selective laser melted meta-biomaterials. J. Mech. Behav. Biomed. Mater..

[5] Attar H., Ehtemam-Haghighi S., Kent D., Okulov I.V., Wendrock H., Bönisch M., Volegov A.S., Calin M., Eckert J., Dargusch M.S. (2017). Nanoindentation and wear properties of Ti and Ti-TiB composite materials produced by selective laser melting. Mater. Sci. Eng. A.

[6] Attar H., Ehtemam-Haghighi S., Kent D., Wu X., Dargusch M.S. (2017). Comparative study of commercially pure titanium produced by laser engineered net shaping, selective laser melting and casting processes. Mater. Sci. Eng. A.

[7] Xia M., Gu D., Yu G., Dai D., Chen H., Shi Q. (2017). Porosity evolution and its thermodynamic mechanism of randomly packed powder-bed during selective laser melting of Inconel 718 alloy. Int. J. Mach. Tools Manuf..

[8] Louvis E., Fox P., Sutcliffe C.J. (2011). Selective laser melting of aluminum components. J. Mater. Process. Technol..

[9] Bremen S., Meiners W., Diatlov A. (2012). Selective laser melting. Laser Tech. J..

[10] AlMangour B., Grzesiak D., Yang J.M. (2016). Rapid fabrication of bulk-form TiB2/316L stainless steel nanocomposites with novel reinforcement architecture and improved performance by selective laser melting. J. Alloys Compd..

[11] AlMangour B., Grzesiak D., Borkar T., Yang J.M. (2018). Densification behavior, microstructural evolution, and mechanical properties of TiC/316L stainless steel nanocomposites fabricated by selective laser melting. Mater. Des..

[12] AlMangour B., Grzesiak D., Yang J.M. (2017). Scanning strategies for texture and anisotropy tailoring during selective laser melting of TiC/316L stainless steel nanocomposites. J. Alloys Compd..

[13] Rashid R., Masood S.H., Ruan D., Palanisamy S., Rahman Rashid R.A., Brandt M. (2017). Effect of scan strategy on density and metallurgical properties of 17-4PH parts printed by Selective Laser Melting (SLM). J. Mater. Process. Technol..

[14] Siddique S., Imran M., Wycisk E., Emmelmann C., Walther F. (2015). Influence of process-induced microstructure and imperfections on mechanical properties of AlSi12 processed by selective laser melting. J. Mater. Process. Technol..

[15] Aboulkhair N.T., Maskery I., Tuck C., Ashcroft I., Everitt N.M. (2016). Improving the fatigue behavior of a selectively laser melted aluminum alloy: Influence of heat treatment and surface quality. Mater. Des..

[16] Prashanth K.G., Scudino S., Klauss H.J., Surreddi K.B., Löber L., Wang Z., Chaubey A.K., Kühn U., Eckert J. (2014). Microstructure and mechanical properties of Al-12Si produced by selective laser melting: Effect of heat treatment. Mater. Sci. Eng. A.

[17] Aboulkhair N.T., Everitt N.M., Ashcroft I., Tuck C. (2014). Reducing porosity in AlSi10Mg parts processed by selective laser melting. Addit. Manuf..

[18] Kempen K., Thijs L., van Humbeeck J., Kruth J.-P. (2015). Processing AlSi10Mg by selective laser melting: Parameter optimisation and material characterization. Mater. Sci. Technol..

[19] Read N., Wang W., Essa K., Attallah M. (2015). Selective laser melting of AlSi10Mg alloy: Process optimisation and mechanical properties development. Mater. Des..

[20] Anwar A.B., Pham Q.C. (2017). Selective laser melting of AlSi10Mg: Effects of scan direction, part placement and inert gas flow velocity on tensile strength. J. Mater. Process. Technol..

[21] Siddique S., Imran M., Rauer M., Kaloudis M., Wycisk E., Emmelmann C., Walther F. (2015). Computed tomography for characterization of fatigue performance of selective laser melted parts. Mater. Des..

[22] Siddique S., Imran M., Walther F. (2016). Very high cycle fatigue and fatigue crack propagation behavior of selective laser melted AlSi12 alloy. Int. J. Fatigue.

[23] Siddique S., Awd M., Tenkamp J., Walther F. (2017). Development of a stochastic approach for fatigue life prediction of AlSi12 alloy processed by selective laser melting. Eng. Fail. Anal..

[24] Siddique S., Awd M., Walther F. Influence of hybridization by selective laser melting on the very high cycle fatigue behavior of aluminum alloys. Proceedings of the 2017 7th International Conference on Very High Cycle Fatigue.

[25] Suryawanshi J., Prashanth K.G., Scudino S., Eckert J., Prakash O., Ramamurty U. (2016). Simultaneous enhancements of strength and toughness in an Al-12Si alloy synthesized using selective laser melting. Acta Mater..

[26] Tang M., Chris Pistorius P. (2017). Oxides, porosity and fatigue performance of AlSi10Mg parts produced by selective laser melting. Int. J. Fatigue.

[27] Zhang H., Zhu H., Qi T., Hu Z., Zeng X. (2016). Selective laser melting of high strength Al-Cu-Mg alloys: Processing, microstructure and mechanical properties. Mater. Sci. Eng. A.

[28] Kaufmann N., Imran M., Wischeropp T.M., Emmelmann C., Siddique S., Walther F. (2016). Influence of process parameters on the quality of aluminium alloy EN AW 7075 using selective laser melting (SLM). Phys. Procedia.

[29] Sistiaga M.L., Mertens R., Vrancken B., Wang X., van Hooreweder B., Kruth J.-P., van Humbeeck J. (2016). Changing the alloy composition of Al7075 for better processability by selective laser melting. J. Mater. Process. Technol..

[30] Wang P., Li H.C., Prashanth K.G., Eckert J., Scudino S. (2017). Selective laser melting of Al-Zn-Mg-Cu: Heat treatment, microstructure and mechanical properties. J. Alloys Compd..

[31] Martin J.H., Yahata B.D., Hundley J.M., Mayer J.A., Schaedler T.A., Pollock T.M. (2017). 3D printing of high-strength aluminium alloys. Nature.

[32] Filatov Y.A., Yelagin V.I., Zakharov V.V. (2000). New Al-Mg-Sc alloys. Mater. Sci. Eng. A.

[33] Davydov V.G., Rostova T.D., Zakharov V.V., Filatov Y.A., Yelagin V.I. (2000). Scientific principles of making an alloying addition of scandium to aluminum alloys. Mater. Sci. Eng. A.

[34] Lathabai S., Lloyd P.G. (2002). The effect of scandium on the microstructure, mechanical properties and weldability of a cast Al-Mg alloy. Acta Mater..

[35] Elagin V.I., Zakharov V.V., Rostova T.D. (1994). Effect of scandium on the structure and properties of alloy Al-5.5% Zn-2.0% Mg. Metal Sci. Heat Treat..

[36] Spierings A.B., Dawson K., Voegtlin M., Palm F., Uggowitzer P.J. (2016). Microstructure and mechanical properties of as-processed scandium-modified aluminium using selective laser melting. CIRP Ann..

[37] Spierings A.B., Dawson K., Heeling T., Uggowitzer P.J., Schäublin R., Palm F., Wegener K. (2017). Microstructural features of Sc- and Zr-modified Al-Mg alloys processed by selective laser melting. Mater. Des..

[38] Singh A., Ramakrishnan A., Dinda G. (2017). Direct Laser Metal Deposition of Al 7050 Alloy.

[39] Singh A., Ramakrishnan A., Dinda G.P., The Minerals, Metals & Materials Society (2017). Direct laser metal deposition of eutectic Al-Si alloy for automotive applications. TMS 2017 146th Annual Meeting & Exhibition Supplemental Proceedings.

[40] Dassault S. (2017). Abaqus 6.12 analysis user guide. Damage and Failure for Ductile Materials in Low-Cycle Fatigue.

[41] Awd M., Siddique S., Tenkamp J., Walther F. (2017). Freeform characterization of fatigue strength of additively manufactured lightweight alloys through FEM and Monte-Carlo modeling. 2. Tagung des DVM-AK Additiv Gefertigte Bauteile und Strukturen.

[42] Prashanth K.G., Shakur Shahabi H., Attar H., Srivastava V.C., Ellendt N., Uhlenwinkel V., Eckert J., Scudino S. (2015). Production of high strength Al85Nd8Ni5Co2 alloy by selective laser melting. Addit. Manuf..

